# Maintaining Genome Integrity: Actin Polymerization Stabilizes Chromatin Bridges in Cytokinesis

**DOI:** 10.3390/ijms27041993

**Published:** 2026-02-19

**Authors:** Sofia Balafouti, George Zachos, Eleni Petsalaki

**Affiliations:** Department of Biology, University of Crete, Vassilika Vouton, 70013 Heraklion, Greece; molgrad417@edu.biology.uoc.gr (S.B.); gzachos@uoc.gr (G.Z.)

**Keywords:** cell division, cytokinesis, chromatin bridges, actin patches, Nesprin-2, RhoA, LINC

## Abstract

In mitotic cell division, cytokinesis is followed by abscission, the final separation of the cytoplasmic canal, to release the two genetically identical daughter cells; however, sometimes chromatin bridges connecting the daughter nuclei appear. Preserving intact chromatin bridges is crucial because their breakage can cause DNA damage, aneuploidy, and cancer predisposition. For this purpose, cells use two main mechanisms: first, they activate the abscission checkpoint, a mechanism that delays the final cut of the cytoplasmic canal to prevent chromatin bridge breakage and secondly, they form accumulations of actin (“actin patches”) at the base of the intercellular canal to stabilize chromatin bridges. Here, we highlight new findings from our laboratory on how human cells “sense” chromatin bridges and remodel the actin cytoskeleton to generate actin patches in cytokinesis. More specifically, we discuss findings showing that the nuclear membrane Sun1/2-Nesprin-2-LINC (linker of nucleoskeleton and cytoskeleton) complex promotes the generation of mechanical tension on daughter nuclei with chromatin bridges. This tension leads to accumulation of Sun1/2 and Nesprin-2, and cytoplasmic accumulation of PDZ RhoGEF (PDZ domain-containing Rho guanine nucleotide exchange factor) at the base of the intercellular canal. In turn, PDZ RhoGEF activates downstream RhoA-ROCK-LIMK-Cofilin and RhoA-mDia1 signaling pathways to promote actin patches and prevent chromatin bridge breakage in cytokinesis.

## 1. Introduction

Chromatin bridges are strands of incompletely segregated chromosomal DNA that link the anaphase poles or daughter nuclei during cytokinesis and can originate from several sources, including improper resolution of DNA recombination intermediates, dicentric chromosomes following telomere fusion, defects in DNA replication, or DNA catenation [1,2]. The presence of chromatin bridges during cytokinesis can lead to tetraploidy or DNA breakage, and has been associated with tumorigenesis [3]. The detrimental effects of chromatin bridge breakage on genomic stability are well documented and include phenomena such as chromothripsis, DNA kataegis, and micronuclei formation [4,5]. Chromothripsis is the fragmentation of one or more chromosomes and their random rearrangement, which often leads to the generation of Tandem Short Template (TST) jumps (short sequences of 200 bases that locate within rearrangement regions) and clusters of point mutations known as kateagis. Chromothripsis appears in many common tumor types and can arise from micronuclei [5]. Micronuclei are acentric chromosome fragments that exhibit a defective nuclear envelope. Due to aberrant surrounding nuclear envelopes, micronuclei are subject to abnormal DNA replication which can lead to extensive DNA damage, integration of their DNA into daughter nuclei, and chromothripsis [5,6].

Approximately 50% of human cancers exhibit chromothripsis, with some cancer types such as soft tissue liposarcomas and bone osteosarcomas exhibiting a prevalence of more than 70–90%, indicating that it can be a key event for cancer development [7,8,9,10]. In cell culture, approximately 30–50% of chromatin bridges resolve after several hours, presumably from a combination of reasons arising from DNA decatenation to chromatin breakage as the daughter cells move apart, to the specific function of nucleases such as Ankyrin Repeat and LEM Domain Containing 1 (ANKLE1) [5,11,12]. Although there is no direct evidence that chromatin bridges cause cancer, a crucial relationship between chromatin bridges and chromothripsis has been established [5] and chromothripsis has been associated with cancer [7,8,9,10].

## 2. Mechanisms of Chromatin Bridge Stabilization

### 2.1. The Abscission Checkpoint

**Figure 1:**
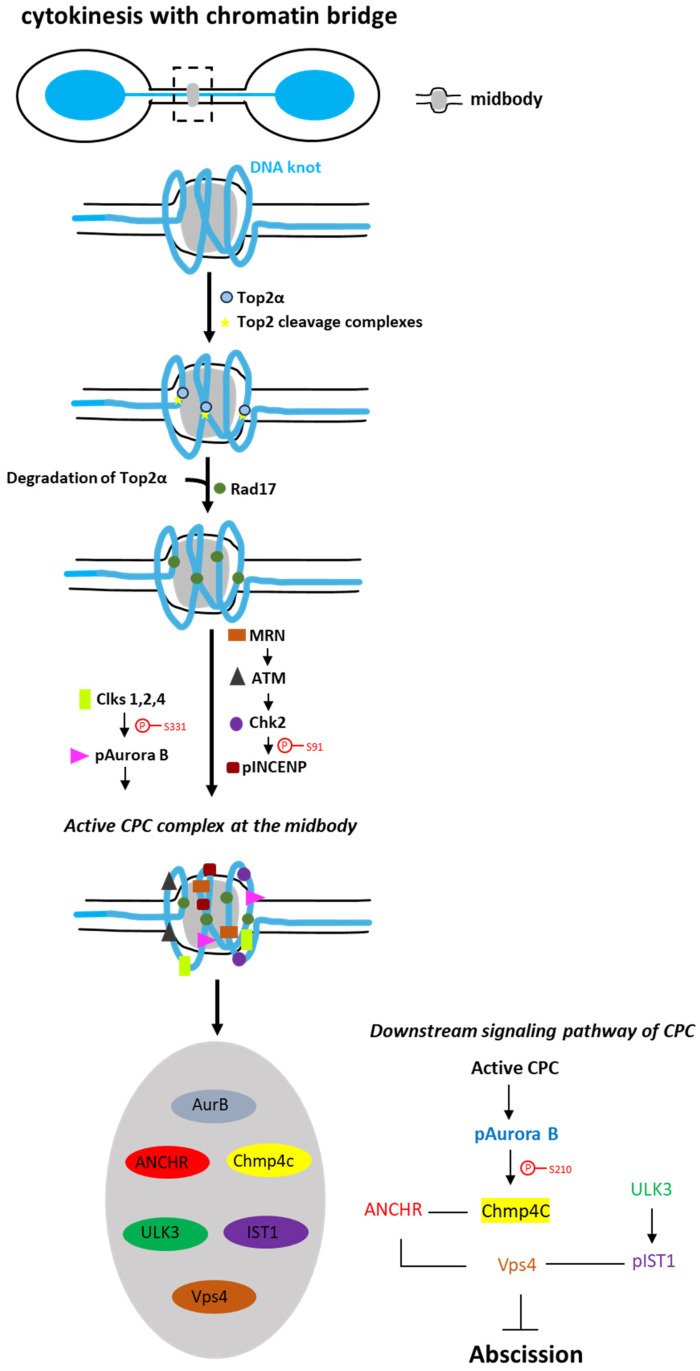
Signaling pathways of abscission checkpoint in cytokinesis with chromatin bridges. Cartoon of a cell undergoing cytokinesis with chromatin bridge. Top2a binds to DNA knots of catenated DNA next to the midbody and recruits Rad17 on DNA knots. In turn, Rad17 is required for MRN localization and activation of downstream ATM-Chk2-INCENP signaling pathway at the midbody. Also, Clks 1, 2 and 4 phosphorylate and activate Aurora B kinase. After the activation of the CPC complex, Aurora B phosphorylates Chmp4c, then ANCHR binds to Chmp4c and Vps4, and ULK3 phosphorylates IST1, which binds to Vps4. In this way, ESCRTIII filament formation is impaired and abscission is delayed.

However, human cells possess mechanisms to stabilize chromatin bridges during cytokinesis. One such mechanism is the activation of the “abscission checkpoint”, which delays the final separation of the intercellular canal, thereby preventing chromatin bridge breakage. This process relies on the activity of the Chromosomal Passenger Complex (CPC) at the midbody [12,13,14]. Recent studies have demonstrated that spontaneous or replication stress-induced chromatin bridges display regions of supercoiled DNA, termed “DNA knots,” near the midbody [15]. The enzyme DNA topoisomerase IIα (Topo2α) binds to these DNA knots, forms abortive Top2 cleavage complexes and facilitates the local recruitment of the adaptor protein Rad17 [16]. Rad17 then recruits the MRN (Mre11-Rad50-Nbs1) protein complex on DNA knots, leading to the activation of an MRN-ATM-Chk2-INCENP signaling pathway that promotes the optimal localization of the CPC complex to the midbody center [16,17]. Cdc-like kinases (Clks) 1, 2 or 4 also localize to the midbody during cytokinesis with chromatin bridges, interact with the catalytic subunit of the CPC Aurora B kinase and phosphorylate Aurora B at Serine 331, which is required for complete activation of Aurora B [18,19]. Clk kinases also localize to the midbody in the absence of chromatin bridges and inhibition of Clk kinases or depletion with siRNAs leads to acceleration of midbody resolution in normally segregating cells [18] (Figure 1).

At the midbody, Aurora B phosphorylates Charged multivesicular body protein 4C (Chmp4c), a component of Endosomal Sorting Complex Required for Transport III (ESCRTIII), in many residues including Serine 210 to implement the abscission delay in response to chromatin bridges [17,18]. Also, in the presence of chromatin bridges, ANCHR (Abscission/NoCut Checkpoint Regulator) localizes at the midbody and binds to ATPase Vacuolar Protein Sorting (Vps4) and Chmp4c generating a complex dependent on Aurora B activity [20]. Furthermore, ULK3 (Unc-51-like kinase 3) kinase localizes at the midbody where it phosphorylates IST1, a subunit of ESCRTIII, that binds to ATPase Vps4 [21]. All the above interactions prevent the formation of ESCRTIII filaments at the secondary abscission site, resulting in abscission delay and stable chromatin bridges. In contrast, dicentric bridges derived from telomere end fusions that do not exhibit knotted DNA do not recruit Top2 next to the midbody; as a result, these bridges fail to activate the abscission checkpoint signaling and break in cytokinesis [16]. Also interestingly, in cells with chromatin bridges induced by replication stress and treated with a Top2α inhibitor, the DNA helicase PARI contributes to the abscission delay through an undescribed mechanism [22] (Figure 1).

How chromatin bridges are resolved is an open question. Maciejowski et al. showed that in cytokinesis with dicentric chromatin bridges, an abscission delay can lead to nuclear envelope rupture during interphase (NERDI) in these cells. NERDI permits access of the cytoplasmic 3′ exonuclease TREX1 across the bridge that generates ssDNA in order to resolve chromatin bridges [4]. Recent research identified a new role of ANKLE1 endonuclease in genome stability. ANKLE1 binds to supercoiled DNA to cleave the DNA intertwines in a controlled manner, preventing chromosomal instability [11].

### 2.2. Formation of Actin Patches

Another mechanism to stabilize chromatin bridges in cytokinesis is the accumulation of polymerized actin at the base of the intercellular canal connecting daughter cells, forming actin-rich structures known as “actin patches” [13,23]. Dandoulaki et al. demonstrated that the DNA damage kinase Chk1 (Checkpoint kinase 1) phosphorylates the nonreceptor tyrosine kinase Src at Serine 51 resulting in its activation, perhaps by promoting an open (active) conformation of the kinase. Inhibition of Chk1 and Src impairs actin patch formation, increases the frequency of broken chromatin bridges and leads to accumulation of DNA damage. Phosphorylated Src and downstream Src-signaling proteins, such as FAK, facilitate actin patch formation to prevent chromatin bridge breakage [23]. Moreover, Chk1 and phosphorylated Src promote the localization of phosphorylated FAK at Tyrosine 925 in actin patches. In a separate study, Bai et al. showed that the methionine sulfoxide reductase B2 MrsB2 is recruited to the midbody, where it interacts with Aurora B and ANCHR, and promotes increased levels of polymerized F-actin in the cytoplasmic canal in the presence of lagging chromatin, to prevent furrow regression and tetraploidy [24]. However, the molecular mechanisms of actin patch formation were incompletely understood.

## 3. The Small GTPase RhoA and Downstream Effectors

RhoA (Ras homolog family member A) is a small GTPase belonging to the RhoGTPase family. RhoA regulates cell polarity, movement, and division by modulating actin and microtubule dynamics [25]. Acting as a molecular switch, RhoA is activated by binding with guanosine triphosphate (GTP) and is deactivated upon binding with guanosine diphosphate (GDP). Guanine nucleotide exchange factors (GEFs) facilitate the exchange of GDP with GTP and activate RhoA, while GTPase activating proteins (GAPs) execute GTP hydrolysis to GDP and inactivate RhoA; as a result, this switching mechanism controls the formation of actin stress fibers and actomyosin contraction. In mammals, more than 70 different RhoGEFs implicated in the activation of RhoGTPases are reported [26]. Some RhoGEFs play a role in the transduction of mechanical signals. Most of these RhoGEFs activate RhoA to promote actin cytoskeleton remodeling in order to respond to mechanical stress [27].

Downstream effectors of RhoA include mDia1 (Diaphanous-related formin 1) and ROCK (Rho-associated protein kinase). mDia1 polymerizes actin to form straight, parallel actin filaments, contributing to cell migration, adhesion, response to mechanical signals, and division [28]. It also binds to RhoA∙GTP via its Rho GTPase binding domain (GBD) [29]. ROCK, on the other hand, is a serine-threonine kinase, which comprises two isoforms ROCK1 and ROCK2, and binds to RhoA∙GTP through its Rho binding domain (RBD) [30,31]. Active ROCK can phosphorylate the myosin light chain (MLC) for actomyosin contraction and can also phosphorylate LIMK1 (LIM domain kinase 1) at threonine 508, activating it. In turn, LIMK1 phosphorylates cofilin, an actin depolymerizing factor, to inactivate cofilin and stabilize preexisting actin filaments [32].

## 4. The LINC Complex

The LINC (Linker of Cytoskeleton and Nucleoskeleton) complex comprises SUN (Sad1p/Unc-84) and KASH (Klarsicht, Anc-1, and Syne Homology) proteins, which interact within the space between the inner and outer nuclear membrane, bridging the cytoplasm with the nucleus [33]. In mammals, five different SUN proteins (SUN1-5), among which SUN1 and SUN2 are widely expressed, and five different genes for KASH proteins (*SYNE1-4*, *KASH5*) have been identified [34]. Proteins encoded by *SYNE1-4* genes are also known as Nesprins (Nuclear envelope spectrin repeats) [35]. Nesprin-2 is a protein of ~800 kD consisting of a C-terminal transmembrane KASH (Klarsicht/ANC-1/SYN3 homology) domain, an N-terminal CH (calponin homology) domain that binds to the actin cytoskeleton and a central region of 56 tandem spectrin repeats (SRs) [36]. The LINC complex is vital for maintaining nuclear morphology, chromosome movement during meiosis, cell migration, organelle anchoring and positioning, and for transmitting mechanical signals from the periphery to the nucleus [37].

## 5. Generation of Actin Patches in Cytokinesis with Chromatin Bridges

**Figure 2:**
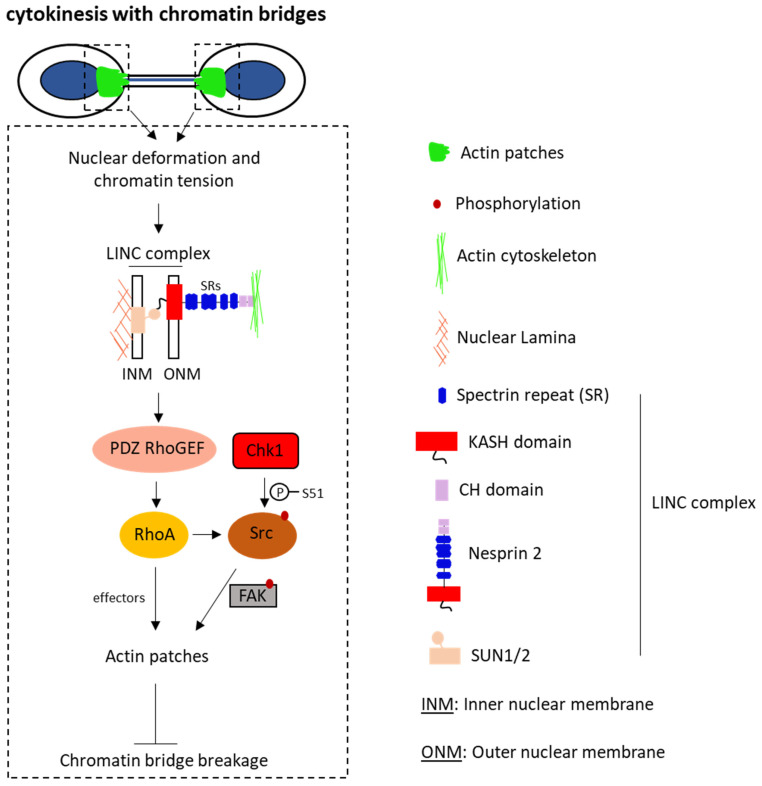
A tension-sensing mechanism promotes actin patch formation in cytokinesis with chromatin bridges. Balafouti et al. [38] show that, in cytokinesis with chromatin bridges, mechanical stress on daughter nuclei (nuclear tension) promotes accumulation of LINC proteins near the base of the intercellular canal and local recruitment of PDZ RhoGEF in the cytoplasm; in turn, PDZ RhoGEF activates RhoA and downstream RhoA–ROCK–LIMK–Cofilin and RhoA–mDia1 signaling (effectors) to generate actin patches. Src and FAK also contribute to actin patch formation downstream of RhoA, perhaps by facilitating actin nucleation and/or by coordinating actin polymerization with actin cytoskeleton remodeling.

Through the analysis of human cancer cell lines using confocal and time-lapse microscopy, Balafouti et al. demonstrated that, in cytokinesis with chromatin bridges, the mechanical tension exerted on daughter nuclei by intact chromatin bridges is converted into biochemical signaling in the cytoplasm via tension-induced activation of a LINC–PDZ RhoGEF–RhoA molecular pathway [38]. The researchers observed RhoA localization at the base of the intercellular canals and its colocalization with actin patches. Depletion of RhoA resulted in reduced actin patches and increased frequency of broken chromatin bridges (Figure 2) [38]. Moreover, membrane labeling revealed that chromatin bridge breakage upon RhoA depletion is not due to premature abscission, but is correlated with reduced actin patches. These findings underscore RhoA’s role in promoting actin patch formation and preventing chromatin bridge breakage in cytokinesis.

## 6. Signaling Pathway During Actin Patch Formation

Furthermore, the researchers identified that RhoA downstream effectors such as ROCK, phosphorylated LIMK at Threonine 508 (pLIMK T508, active), phosphorylated Cofilin at Serine 3 (pCofilin S3, inactive), and mDia1 localize to actin patches in a RhoA-dependent manner, indicating a direct role for these proteins in actin patch formation [38]. They showed that inhibition of ROCK, LIMK, and mDia1 is associated with reduced actin patches and increased chromatin bridge breakage during cytokinesis. Through inhibition of RhoA-signaling proteins by small molecule inhibitors in various drug combinations, the authors showed that mDia1 collaborates with the ROCK–LIMK–Cofilin signaling pathway downstream of RhoA to prevent chromatin bridge breakage [38]. Also, expression of constitutively active Src-S51D or FAK-Y397E bearing phosphomimetic mutations of key regulatory residues rescued actin patches and prevented chromatin bridge breakage in RhoA-deficient cells, indicating that RhoA acts upstream of Src and FAK during actin patch formation [38]. They also showed that PDZ RhoGEF is essential for stable chromatin bridges, that PDZ localizes to actin patches and that localization of RhoA at the base of the intercellular canal is PDZ-dependent [38]. These results suggest that PDZ RhoGEF activates RhoA to promote actin patch formation in cytokinesis with chromatin bridges.

## 7. Sensing of Chromatin Bridges

To explore how human cells “sense” chromatin bridges to induce actin patch formation, the authors examined the shape of nuclear chromatin or nuclear lamina by high-resolution confocal microscopy. They observed deformed (oval-shaped) nuclei in cells with intact chromatin bridges, compared with interphase cells without chromatin bridges exhibiting an approximately round shape [38]. Additionally, cells with intact chromatin bridges displayed “nuclear lines” of Sun1, Sun2, and Lamin A indicating deformed nuclear membrane. Collectively, the above changes in nuclear shape and membrane indicate that cells with chromatin bridges are under mechanical tension [39,40,41]. Furthermore, the authors showed that during cytokinesis with intact chromatin bridges, Sun1, Sun2, and Nesprin-2 proteins of the LINC complex accumulate towards the base of chromatin bridges, close to actin patches [38]. These results suggest that pulling forces exerted on daughter nuclei by intact chromatin bridges induce nuclear stress and the accumulation of the Sun1/2-Nesprin-2 LINC complex at the base of the chromatin bridge.

Balafouti et al. also showed that depletion of LINC proteins, but not RhoA, correlates with reduced nuclear shape deformation and diminished nuclear lines. Furthermore, disruption of the LINC complex is associated with impaired actin patches and broken chromatin bridges in cytokinesis [38]. Together, these findings establish a previously unrecognized connection between the LINC complex, nuclear tension, and actin patch formation.

## 8. Interaction Between Nesprin-2 and PDZ

To further elucidate the role of the Sun1/2-Nesprin-2 LINC complex in actin patch formation, the researchers employed mutant mouse mini-Nesprin proteins CB and CH* that lack spectrin repeats (SRs) 3-54. The CB protein retains the ability to bind to the actin cytoskeleton through its CH domain, whereas CH* lacks this ability due to mutations in its CH domain [41,42]. By employing these proteins in siRNA depletion–replacement experiments, the researchers established that SRs 3-54 of Nesprin-2 are required for actin patch formation and stable chromatin bridges in cytokinesis. Furthermore, they demonstrated that SRs 3-54 interact with PDZ RhoGEF in cell extracts and in vitro. They also showed that in cells with intact chromatin bridges in cytokinesis, interaction of Nesprin-2 with the actin cytoskeleton and an intact nuclear lamina are required for nuclear tension and accumulation of LINC complex near the base of chromatin bridges [38].

To further explore the significance of the Nesprin-2–PDZ interaction during cytokinesis with chromatin bridges, the researchers targeted RhoA to the cytoplasmic region of Nesprin-2. Specifically, they inserted SRs 31-37, which interact with PDZ RhoGEF, or the DH/PH domain of PDZ that interacts with RhoA into the mini-Nesprin CB or CH* proteins, generating CB:SRs31-37, CB:DHPH, or CH*:DHPH proteins. They found that expression of these proteins rescues actin patches and reduces chromatin bridge breakage in Nesprin-2-deficient cells only when mini-Nesprin is associated with the actin cytoskeleton [38]. This indicates that, in the presence of nuclear tension, targeting RhoA to the Nesprin-2 cytoplasmic region promotes actin patch formation and prevents chromatin bridge breakage during cytokinesis.

## 9. The Role of Nesprin-2 in Actin Patch Formation

The researchers also showed that in cells with intact chromatin bridges, mini-Nesprin accumulates at the base of chromatin bridges only in the presence of mechanical tension, indicating a correlation between nuclear tension and Nesprin-2 accumulation at the base of chromatin bridges during cytokinesis. They then explored the potential role of Nesprin-2 enrichment for actin patch formation. First, they demonstrated that Nesprin-2 was mislocalized to nuclear granules after overexpression of Lamin A, preventing tension-induced actin patch formation and promoting the formation of actin foci that colocalize with Nesprin-2 specifically through RhoA signaling [38]. Second, they showed that increased nuclear stiffness reduced Nesprin-2 accumulation at the base of chromatin bridges and decreased actin patch formation [38]. These findings suggest that Nesprin-2 enrichment at the base of chromatin bridges promotes actin patch formation.

## 10. Conclusions and Outstanding Questions

**Figure 3:**
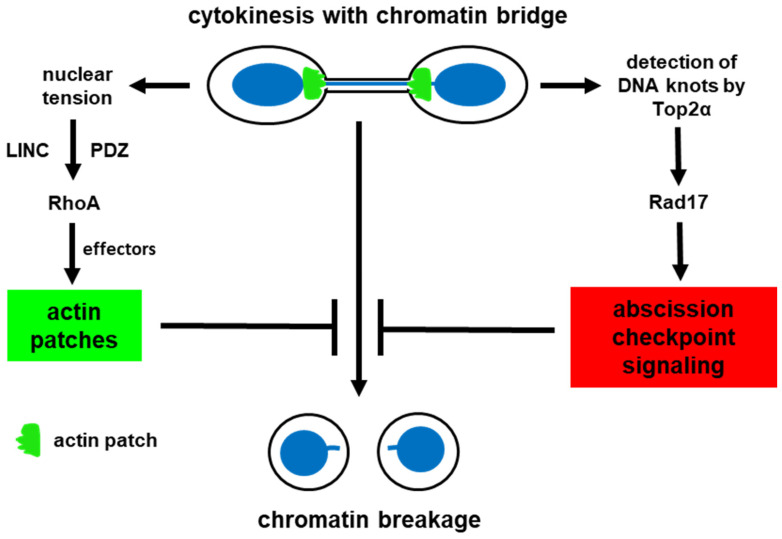
Chromatin bridge-sensing mechanisms that induce actin patch formation and delay abscission in human cells. In cytokinesis with chromatin bridges, mechanical stress on daughter nuclei (nuclear tension) is transmitted to the cytoplasm through the LINC complex to activate PDZ RhoGEF–RhoA signaling and to generate actin patches. On the other hand, DNA “knots” on chromatin bridges with catenated DNA are detected by Top2α, which forms abortive cleavage complexes on DNA knots [16]. These complexes recruit Rad17 and activate downstream MRN-ATM-Chk2-CPC signaling at the midbody to delay abscission. Together, the above two mechanisms prevent chromatin bridges from breaking in cytokinesis.

The findings by Balafouti et al. are the first to describe a mechanism by which human cells detect chromatin bridges to generate actin patches in cytokinesis. They show that mechanical tension exerted on daughter nuclei by chromatin bridges through the LINC complex promotes the accumulation of Nesprin-2 towards the base of chromatin bridges and facilitates PDZ RhoGEF accumulation, through PDZ-binding to Nesprin-2 cytoplasmic spectrin repeats. In turn, PDZ RhoGEF locally activates RhoA-ROCK-LIMK-Cofilin and RhoA-mDia1 signaling pathways to promote actin patch formation and prevent chromatin bridge breakage during cytokinesis [38]. Together with the recent identification of chromatin bridge sensing by Top2α, through Top2α binding to DNA knots, which activates abscission checkpoint signaling to delay abscission [16], these findings describe novel mechanisms that stabilize chromatin bridges in cytokinesis and prevent them from breaking (Figure 3).

Previously established mechanotransduction pathways transfer mechanical forces from the cell periphery to the nucleus to rearrange chromatin and modify gene expression [35]. However, the mechanism described by Balafouti et al. [38] operates “inside-out” by responding to endogenous stress to regulate actin cytoskeleton. Because chromatin bridge breakage is associated with chromothripsis, DNA kataegis, and micronuclei formation in various cancers, this mechanism contributes to genome integrity and could safeguard against cancer.

## 11. How Do Actin Patches Protect Against Chromatin Breakage?

This study also raises several important questions. One possibility is that actin patches absorb the forces generated by continuous pulling of bridged chromosomes, thus preventing DNA breakage. Alternatively, actin patches could increase cell stiffness to provide mechanical support, similar to nuclear actin polymerization in migrating cells [32,43]. It would perhaps be interesting to examine whether/how soft versus stiff substrates influence tension-induced actin patch formation in cytokinesis with chromatin bridges. Is stretched DNA the only mechanical factor that induces actin patch formation? Or are there other circumstances in which actin patches can be formed within the cell, for example, in the presence of tension inside the intercellular canal without chromatin bridges [12]? Do chromatin bridges that are processed by the endonuclease ANKLE1 form actin patches or do ANKLE1 cut chromatin bridges in a controlled way in the absence of actin patches?

Also, how does nuclear tension promote enrichment of Nesprin-2 toward the base of chromatin bridges? Is it because membrane deformation by bridge forces is perhaps stronger near the base of the chromatin bridge, or is it through some active mechanism that transports LINC complexes?

It was previously shown that dicentric chromosomes without knotted DNA do not activate the spindle checkpoint [16], making this type of bridge more vulnerable to DNA breakage. Could these bridges form actin patches? Because dicentric bridges are predicted to cause deformations of daughter nuclei, similar to spontaneous bridges from catenated chromosomes, it is possible that dicentrics generate actin patches. If true, actin patches could be very important for protecting older tissues, where dicentric bridges are relatively more frequent due to telomere attrition, from carcinogenesis. It was previously shown that depletion of Top2a in centromeric chromatin bridges leads to chromatin breakage with persistent actin patches [16]. Uncoupling abscission checkpoint signaling from actin patch formation could ensure that, if one mechanism malfunctions, the other can still provide a level of protection against chromatin bridge breakage in cytokinesis. However, some level of cross-talk, which could coordinate these two events in cytokinesis with catenated bridges, cannot be excluded. Perhaps interestingly, PARI helicase that contributes to abscission checkpoint activation is also required for persistent actin patches in human cells [22]. What happens in cells in cytokinesis that are under mechanical tension and exhibit no chromatin bridges inside the cytoplasmic canal? Finally, can actin patches be exploited for cancer therapy? For this purpose, it will be important to investigate whether interfering with actin patch formation by small molecule inhibitors against actin patch regulators can selectively kill cancer cells with chromatin bridges or prevent them from proliferating. The discovered mechanism that converts mechanical stress exerted on daughter nuclei from chromatin bridges to reorganization of the actin cytoskeleton will be essential in answering these important questions in the future.

## Data Availability

No new data were created or analyzed in this study. Data sharing is not applicable to this article.
